# Neural Plasticity in Multiple Sclerosis: The Functional and Molecular Background

**DOI:** 10.1155/2015/307175

**Published:** 2015-07-02

**Authors:** Dominika Justyna Ksiazek-Winiarek, Piotr Szpakowski, Andrzej Glabinski

**Affiliations:** Department of Neurology and Stroke, Medical University of Lodz, Zeromskiego Street 113, 90-549 Lodz, Poland

## Abstract

Multiple sclerosis is an autoimmune neurodegenerative disorder resulting in motor dysfunction and cognitive decline. The inflammatory and neurodegenerative changes seen in the brains of MS patients lead to progressive disability and increasing brain atrophy. The most common type of MS is characterized by episodes of clinical exacerbations and remissions. This suggests the presence of compensating mechanisms for accumulating damage. Apart from the widely known repair mechanisms like remyelination, another important phenomenon is neuronal plasticity. Initially, neuroplasticity was connected with the developmental stages of life; however, there is now growing evidence confirming that structural and functional reorganization occurs throughout our lifetime. Several functional studies, utilizing such techniques as fMRI, TBS, or MRS, have provided valuable data about the presence of neuronal plasticity in MS patients. CNS ability to compensate for neuronal damage is most evident in RR-MS; however it has been shown that brain plasticity is also preserved in patients with substantial brain damage. Regardless of the numerous studies, the molecular background of neuronal plasticity in MS is still not well understood. Several factors, like IL-1*β*, BDNF, PDGF, or CB1Rs, have been implicated in functional recovery from the acute phase of MS and are thus considered as potential therapeutic targets.

## 1. Introduction

Multiple sclerosis (MS) is a chronic autoimmune disease of the central nervous system (CNS) which leads to demyelination and subsequent neurodegeneration. It usually affects young adults, with disease onset occurring between 20 and 40 years of age. The clinical signs and disease course of MS are heterogeneous and depend on the brain region affected [1]. Although the exact cause of the disease remains unknown, the role of the immune system in its development is evident. The onset and progression of MS are linked to numerous inflammatory processes that occur in various parts of the CNS. White matter infiltration by immune cells is the major hallmark of MS [2]. The key immune players responsible for the CNS inflammation seen in MS are CD4-positive T lymphocytes; however, other types of inflammatory cells like monocytes/macrophages, neutrophils, and B lymphocytes are also involved. Infiltrating cells secrete a variety of factors that modulate neuronal function and signal formation in neuronal synapses, thereby affecting brain plasticity. Cellular and secretory activity of infiltrating leukocytes contribute to the formation of demyelinated lesions in the white matter, with inflammatory foci and neuronal damage, which in consequence lead to the presence of clinical symptoms [3]. The gray matter of patients with MS is also affected, leading to motor, sensory, visual, and cognitive impairment. In fact, about half of MS patients suffer from a decrease in cognitive functions, such as memory and learning abilities [3].

It was long believed that the human brain did not change substantially after the initial phase of development. However, it is now widely accepted that the brain demonstrates structural and functional plasticity throughout life, allowing it to cope with everyday challenges. By the induction of various mechanisms that modify neural pathways and synapses, the brain can adapt dynamically to everyday environmental and pathological stimuli, which is defined as neuronal plasticity. The basic, physiological role of this process is related to brain development, learning, and memory [4–7]. In a pathological condition, neuronal plasticity is engaged in the healing process of brain injuries. The term “neuroplasticity” encompasses a wide range of changes, such as the altered strength of synaptic transmission, the formation of novel synapses, cortical reorganization, and the induction of neurogenesis. An example of brain plasticity includes changes in the equilibrium of excitation and inhibition [8]. It is known that neurons are interconnected throughout a larger anatomical area than that which they functionally influence. Abolition of inhibitory interactions may increase their territory of functioning [8]. Another example is the changes in the membrane voltage-gated ion channels, leading to modulated neuronal membrane excitability [9]. Alterations in synaptic efficacy are also a form of neuronal plasticity. Depending on the type of stimuli, existing synapses may be strengthened or weakened leading to induction of long-term potentiation (LTP) or long-term depression (LTD), respectively [10, 11]. The mechanisms leading to structural reorganization are not fully recognized. It has been shown, however, that, during cortical reorganization, the sprouting of axons, the formation of new synapses, and the reinduction of certain developmental programs occur [12].

Elements of innate and adaptive immunity have a significant impact on structural and functional plasticity in neuropathological conditions, as they can both favor and hamper brain recovery [13]. The failure of CNS plasticity may result in a more pronounced susceptibility to chronic stress-mediated diseases, psychopathologies, and neurodegenerative disorders.

The neuropathological hallmarks of MS are multifocal inflammation, demyelination, and neurodegeneration [14, 15]. Remyelination is a crucial process for repairing inflammatory demyelinated lesions [16]; however, adaptive plasticity has been shown to be responsible for clinical recovery [17–19]. This suggests that the brain's functional adaptive reserve is still active in MS. Yet this capacity of the brain to manage accumulating damage differs between patients and between various disease types, leading to large interpersonal divergence. Moreover, neuronal plasticity decreases with a patient's age and with the length of disease duration [20, 21]. Results from several studies indicate that physical activity and various forms of mental training may accelerate the ability of the brain to slow down clinical progression, even in patients with substantial brain damage. These findings have made neuronal plasticity and personalized neurorehabilitation the focus of recent neuroscientific research with the identification of new therapeutic targets [22, 23].

## 2. fMRI and Brain Plasticity in Multiple Sclerosis

Functional MRI (fMRI) is a relatively new method of brain function analysis which allows for the acquisition of information about the brain's development, physiology, and pathology. fMRI has also become a powerful and promising tool for the study of brain plasticity and its role in many neurological diseases. This method indirectly shows the activation of specific brain regions by demonstrating an increased blood flow to where oxygen-rich blood is needed by aroused neurons. There are several studies suggesting that brain plasticity can compensate for the disseminated brain injury observed in MS. This plasticity may be present locally at the site of injury (synaptic reorganization) or may involve distant uninjured brain regions and pathways. The presence of brain plasticity in MS may explain the common lack of correlation between conventional brain MRI findings and the clinical disability observed in MS patients.

Longitudinal brain fMRI analysis revealed more extended bilateral motor activation in MS than in controls [19]. In this study, fMRI was performed twice and brain activity was stimulated by finger opposition movements. These changes may represent compensatory mechanisms that maintain normal brain function in patients with damaging diseases like MS. In normal controls, the response is mostly contralateral. Increased ipsilateral motor cortex activation suggests that in MS hemispheric lateralization is decreased. Patients with milder brain damage demonstrated more lateralized brain activity. In patients with more severe disease activity, the tendency towards lateralization of brain function, which is typical for healthy controls, was arrested [19]. In another study, cortical motor activation in the ipsilateral sensorimotor cortex of MS patients correlated inversely with axonal injury measured by magnetic resonance spectroscopy (MRS). These observations confirm that cortical plasticity may lead to decreased hemispheric lateralization in MS [24]. Hemispheric lateralization is typical for healthy controls and the concentration of N-acetylaspartate (a marker of axonal integrity) in the brain correlates with an increasing lateralization index. This suggests that rising lateralization is helpful for MS patients. The contralateral premotor cortex of MS patients is more involved in controlling specific movements than that of controls, as shown by fMRI demonstrating its higher activation which was not diminished by training [25]. Cognitive functions are often impaired in MS patients, even at the beginning of the disease. The distribution of cortex activation detected by fMRI during an attention task was evidently different in MS patients compared to controls. This is a similar observation to what is seen in motor task studies, suggesting the presence of brain plasticity during early MS [26]. Another interesting observation in this field was the analysis of the influence of cardiorespiratory fitness on brain plasticity in MS patients. Higher fitness levels correlated with better behavioral data, like reaction time and accuracy. fMRI scanning showed that participants with a higher fitness level had increased brain activation in the same cerebral cortex region that is typically activated in MS patients while performing the PVSAT (Paced Visual Serial Addition Test). Activation of this area was not observed in low-fit patients. This study suggests that aerobic training may be helpful in stimulating neuronal plasticity in patients with MS [27].

Long-term potentiation (LTP) is one of the most important and most studied forms of synaptic plasticity. It is related to experience-dependent changes in CNS function, such as memory or learning [24, 28]. LTP results in intensified communication between simultaneously excited neurons, leading to enhanced synaptic transmission. Thus, it requires the cooperation between pre- and postsynaptic neurons. Results from various studies have pointed to the important role of LTP in plasticity of synaptic morphology. LTP induction may result in an increased size and shape of dendritic spines, promotion of their clustering, and also the growth of dendrites [25–27, 29]. As such, LTP may restore excitation in denervated neurons or in those lacking part of their synaptic inputs [17, 30]. LTP is preserved in relapsing-remitting MS (RR-MS) patients and plays an important role in recovery from neurological deficits. It was suggested that a more efficient LTP response examined during relapse correlated with a better clinical recovery in RR-MS patients, observed as a low or null change in EDSS (Expanded Disability Status Scale) [31]. LTP is present in stable MS patients, while it is ineffective in the progressive form of the disease [31]. Both cTBS (continuous theta-burst stimulation) and iTBS (intermittent theta-burst stimulation) protocols did not induce plastic changes of cortical excitability in MS patients with the progressive form of the disease. It was then suggested that primary-progressive MS (PP-MS) patients have lost their potential to induce synaptic plasticity and, thus, to mask the clinical progression of the disease [32]. Rossi et al. have indicated that a single nucleotide polymorphism (SNP) of the NMDA receptor (rs4880213 allele T) is associated with the increased synaptic transmission [33]. However, this genetic variant exerts opposite effects on PP-MS and RR-MS patients. In PP-MS, it leads to exacerbated excitotoxicity and clinical worsening, while in RR-MS it was associated with an improved clinical outcome due to more efficient LTP [33].

In the striatum and cerebellum of mice with experimental autoimmune encephalomyelitis (EAE), an animal model of MS, increased glutamatergic transmission and decreased GABAergic transmission were present [34–36]. Nisticò et al. indicated that spontaneous release of GABA was significantly reduced in EAE mice, and a lower number of GABAergic interneurons were also observed, leading to the assumption that the vulnerability of these neurons is the cause of synaptic hyperexcitability seen in EAE [35, 37]. These results are in accordance with findings from MS patients showing selective loss of parvalbumin-positive (PV-+) GABAergic interneurons and reduced neurites of PV-+ neurons in the normal appearing gray matter and frontal cortex [38, 39]. Such altered equilibrium of excitation and inhibition may result in more pronounced LTP; however, in the case of considerable dysfunction of GABAergic transmission, it may lead to excitotoxicity, as seen in PP-MS patients. Taken together, it is evident that LTP expression can minimize the outcome of neuronal damage present in MS, which eventually results in the masking of the clinical progression of the disease.

Overall, recent clinical and scientific findings indicate that brain plasticity is responsible for various degrees of functional recovery in MS patients and that the capability of the CNS to manage the neuronal damage is partially regulated by various prosurvival molecules. In the next section, we will review the most important molecular factors regulating LTP occurrence in MS patients.

## 3. Molecular Regulators of Neuronal Plasticity in MS

The concept that the immune system may influence neuronal plasticity is quite recent, as for many years the CNS was considered as an immune privileged organ. Studies from the past two decades have shed some light on the reciprocal interactions between CNS and the immune system. An example of such bidirectional cooperation may be the so-called “sick behavior.” During infectious diseases, when the peripheral immune response is active, CNS functions are altered and social or psychological activity is affected. Sick behavior is mediated by an increased level of proinflammatory cytokines secreted by immunocompetent cells [40, 41]. In contrast, acute stroke patients with damage to the CNS have an increased risk of infections together with decreased immune system efficacy [41–43]. Thus, we want to present recent knowledge about the most prominent immune- and CNS-related molecules shaping the neural plasticity in MS.

### 3.1. BDNF and Neural Plasticity

One of the most important regulators of neuronal plasticity is the neurotrophin called brain derived neurotrophic factor (BDNF). BDNF supports neuron survival and the rearrangement of the cytoskeleton (processes important for the creation of new neuronal synapses) by the upregulation of gene expression and protein synthesis. What is interesting, brain-localized inflammation may lead to an increase in BDNF concentration, providing a neuroprotective effect.

In physiological conditions, BDNF secretion promotes the generation of long-term potentiation (LTP); thus, BDNF is important for long-term memory formation [44, 45]. Molecular pathways underlying the neuroplastic effect of BDNF include the facilitation of F-actin formation. Studies on various animal models have confirmed the impaired LTP generation in the hippocampus of either BDNF or TrkB (receptor for BDNF) deficient mice [46, 47]. In the brain, neurons and activated astrocytes are considered as a major source of BDNF. However, activated lymphocytes such as T-cells and B-cells may also secrete this neurotrophin [48–51]. In brain tissue affected by inflammation, BDNF is released from activated astrocytes, infiltrating B-and T-cells. BDNF secretion may be considered as a protective mechanism, preventing neurons from cell death during inflammatory reactions localized in the brain, due to the fact that BDNF provides trophic support for the development of cholinergic, GABAergic, serotonergic, and dopaminergic neurons [51–55]. Numerous studies have also shown a decreased level of serum BDNF in MS patients, compared to healthy donors [56]. In addition, a decrease in BDNF secretion from lymphocytes may be thought to be linked with disease conversion to the secondary progressive form. In RR-MS, BDNF secreted from lymphocytes may promote neuroplasticity mechanisms for compensation of inflammation-induced cell death in brain. For example, BDNF as a neurotrophin may provide a signal for differentiation of neuronal progenitor cells located in the brain. In* in vitro* studies, BDNF was also shown to mediate neuron myelination in an NMDA receptor-dependent manner [57]. It is unknown why as the disease progresses the phenotype of lymphocytes changes and they stop secreting BDNF. As the generation of autoreactive T-cells depends on the functions of professional antigen presenting cells, it is reasonable to determine conditions that drive dendritic cells toward a functional phenotype inducing BDNF secretion in activated T-cells. These findings could be helpful for prolonging and even enhancing the neuroprotective effects of inflammation and may form the basis for development of new immunomodulating therapies.

### 3.2. IL-1*β* and Neural Plasticity

IL-1*β* is one of the most widely described immune system signal molecules affecting CNS functioning. It belongs to the IL-1 cytokine family that possess proinflammatory and pyrogenic properties. Its secretion occurs in various types of immune cells, but its major secretors are CD4-positive lymphocytes, monocytes, macrophages, and dendritic cells, as well as nonimmune cells like keratinocytes. What is interesting is that, under physiological conditions, IL-1*β* plays a regulatory role in the CNS. It was found in animal models that the concentration of IL-1*β* in the hippocampal area of the brain increased during learning and is important for memory consolidation [41, 58]. However, activation of the immune system due to the peripheral infection dramatically increases the level of IL-1*β* in the brain and leads to cognitive decline [59–61]. These findings may suggest a dose-dependent mode of IL-1*β* activity. In low concentrations, IL-1*β* seems to be important for hippocampal activity but a transient increase of IL-1*β* was found to impair hippocampus mediated memory processing [62, 63].

In MS, brain infiltrating activated immune cells are a potent source of IL-1*β* which lead to its concentration exceeding normal physiological levels. Studies conducted in EAE models of MS confirmed the impact of the immune system on synaptic transmission and plasticity. Numerous studies have described inflammation-driven alterations in neuronal plasticity associated with changes in the LTP/LTD ratio [3, 37, 41, 64–66]. In high concentrations, IL-1*β* is able to lower the threshold for LTP generation. The exact explanation for this action remains unknown; however, studies on EAE mice have shown that hippocampus-infiltrating T lymphocytes release IL-1*β* and thereby promote LTP over LTD, most likely by the suppression of GABAergic transmission and the promotion of glutamatergic transmission with NMDA-mediated Ca^2+^ influx [32]. Such an effect was not observed in T-cells from control mice [32]. In patients that suffer from RR-MS, IL-1*β* levels in the cerebrospinal fluid (CSF) have been correlated with a greater susceptibility to LTP-like synaptic phenomenon induced by theta-burst transcranial magnetic stimulation (TBS), whereas induction of LTD-like synaptic phenomenon proved ineffective [32]. The exact role of IL-1*β* in neuronal plasticity is not fully understood.* In vitro* and* in vivo* studies frequently present contradictory data. The final effect of IL-1*β* seems to be dependent on cytokine concentration, brain region, and the interplay of other factors like BDNF or microglia cells. It could be assumed that the effect of IL-1*β* is a result of its interactions with various cellular factors and its soluble molecular milieu.

### 3.3. IL-1*β* Suppresses BDNF Signaling

Another mode of IL-1*β* action is the intracellular blockade of the BDNF signaling pathway. BDNF is a neurotrophin important in CNS development and proper functioning and neuromediator release and for neuronal survival after damage [54, 67]. Tong and coworkers have proposed the possible mechanism for IL-1*β* action. Using rat hippocampal cultures, they found that IL-1*β* suppresses the BDNF-dependent regulation of* Arc* (critical for memory and learning processes) and phosphorylation of cofilin (actin-binding protein involved in reorganization of actin filaments) and CREB (transcription factor regulating* Arc*). Authors found that IL-1*β* acts on BDNF signal transduction through the upregulation of p38 MAPK [68]. It is possible that similar effects of IL-1*β* occur in the brains of MS patients and that a high concentration of IL-1*β* abolishes the neuroprotective effect of BDNF.

### 3.4. IL-1*β*-Mediated Microglial Activation

Brain-localized inflammation may also lead to IL-1*β*-mediated microglial activation. Microglia are the resident innate immune cells of the CNS. Their main role is the fast response to invasion of infectious agents, in particular, bacteria. However, microglial cells may also phagocytose cellular debris. Moreover, microglia mediate the repair of many pathological processes in the CNS [69].

Microglial cells are able to directly interact with neurons and are considered to be regulators of neuronal plasticity. Under physiological conditions, microglia exhibit a quiescent state of activation. Various stimuli (pathogens, pathogen-associated molecular patterns, and cellular debris) and a cytokine milieu induce microglial activation towards a specific functional phenotype. Generally two phenotypes of activated microglia can be distinguished: M1 (classical) and M2 (alternative). The direction of microglial activation (M1 or M2) depends on the activating milieu (stimulus and cytokine environment). The classical M1 phenotype is important in the fight against infectious agents. M1 microglia secrete a variety of proinflammatory cytokines like IL-1*β*, IL-6, and TNF-*α* and also produce high amounts of reactive oxygen/nitrogen species. M2 microglia secrete anti-inflammatory cytokines like IL-10 and TGF-*β* and exhibit neuroprotective activity. Moreover, microglia with the M2 phenotype produce insulin-like growth factor 1 (IGF1) and PDGF, secrete phosphoprotein 1 (SPP1), and support remyelination [70, 71]. Prolonged activity of M1 microglia and lack of conversion from the M1 to M2 phenotype or an inefficient number/response of M2 microglia may lead to the development of neurodegeneration [70, 72]. In MS, the proinflammatory environment provided by IL-1*β* and other cytokines like IFN-*γ* or TNF-*α* activates brain microglia toward the M1 phenotype and induces the production of additional amounts of IL-1*β* as specific feedback of IL-1*β*-microglia interactions. Thus, the effect of IL-1*β* on neuronal plasticity is further facilitated.

### 3.5. The Role of Amyloid-*β*_1–42_ in MS Neural Plasticity

LTP in MS patients may be altered by various factors. It was observed that excessive production of reactive oxygen species (ROS) can impair the LTP in the hippocampus. One of the molecules inducing increased ROS generation is oligomeric amyloid-*β* [73]. Although A*β* is the hallmark of Alzheimer's disease (AD), it is also observed in multifocal MS lesions [74, 75]. Moreover, the presence of A*β* is associated with altered synaptic plasticity and cognitive functions, observed both in AD and in MS.

Amyloid-*β* (A*β*) peptides are derived from the amyloid precursor protein (APP), which undergoes proteolytic cleavage by *β*-secretase and *γ*-secretase [76]. Such proteolytic processing may result in two A*β* species: A*β*_1–42_, which is more toxic and more prone to aggregation, and A*β*_1–40_, which is thought to be less pathogenic [77]. A*β* is a marker for the neurodegenerative process, with an important role in cognitive and synaptic dysfunctions which has been recognized mainly by Alzheimer's disease preclinical and clinical studies. Its role in MS is only marginally understood.

A*β* has been shown to inhibit hippocampal NMDA-dependent LTP and to facilitate LTD [78, 79]. It is able to disrupt both early and late phases of LTP [80, 81]. A*β* may also alter hippocampal LTP by the reduction of AMPA receptors' currents [82]. Thus, it is suggested that A*β* reveals synaptotoxicity through synapse desensitization or internalization of glutamate receptors and also by interactions with glutamate transporters [79, 83–85]. Moreover, it was shown that A*β* dimers obtained from AD patients induce dendritic spine retraction in mouse neurons and block the induction of LTP, resulting in cognitive decline [86]. Additionally, it has been reported that A*β* inhibits hippocampal LTP by induction of endogenous TNF-*α* release and activation of TNF-R1 [87].

There is conflicting data regarding the role of A*β* in the EAE immune pathology. Furlan et al. have shown that immunization with A*β*_1–42_ leads to symptoms and pathology similar to those seen in EAE, whereas Grant et al. and Kurnellas et al. have shown the anti-inflammatory effect of A*β*_1–42_ or amyloid fibrils, resulting in reduced pathology and diminished symptoms [88–90]. It was also indicated that mice lacking APP have a more serious clinical course [89]. Data from the studies conducted on MS patients are contradictory as well. Some authors have observed elevated or unchanged levels of A*β*_1–42_ in the CSF, without any significant correlation with age or disease duration [91–93]. Others have investigated reduced A*β*_1–42_ content in the CSF of MS patients [94, 95]. Such discrepancies, at least in part, may be due to the various forms of soluble A*β* present in the CSF. Mori et al. have shown that CSF concentrations of A*β*_1–42_ were lower in gadolinium-enhanced (GD^+^) MS patients compared to GD^−^ or non-MS patients [96]. A*β*_1–42_ was also correlated with cognitive deficits, as a reduced concentration of this peptide was present in patients with cognitive decline [96]. It was observed that a decrease in the level of CSF A*β*_1–42_ was associated with alterations in the specific cognitive domains, mainly those connected with attention, concentration, and information-processing speed, the same ones which were altered by the presence of radiologically active lesions [96, 97]. CSF content of A*β*_1–42_ was positively correlated with LTP amplitude, suggesting its regulatory role in memory-related synaptic plasticity, observed both in MS and in EAE [86]. It was suggested that acute inflammatory process disturbs A*β* metabolism and leads to cognitive deficits by altering LTP-like activity-dependent synaptic plasticity [96].

The correlation between CSF levels of A*β*_1–42_ and cognitive decline was observed already in the early phases of AD [98]. It was found that in the CSF of AD patients the concentration of A*β*_1–42_ is rather low. This can be explained by the presence of elevated A*β* deposition in brain plaques or its oligomerization in the CSF [99, 100]. Whether this is also true for MS is currently not known and requires further investigation.

### 3.6. The Role of Platelet-Derived Growth Factor (PDGF) in MS Plasticity

It is well known that various immune cells, especially T-cells, accumulating in the CNS of MS patients contribute to tissue damage and, as a result, to disease progression [101]. However, these cells equally play an important role in the protective mechanisms that result in disease remission. Among a plethora of released molecules, immune cells secrete growth factors such as platelet-derived growth factor (PDGF), fibroblast growth factor (FGF), granulocyte colony-stimulating factor (G-CSF), and granulocyte-macrophage colony-stimulating factor (GM-CSF) [102, 103]. Growth factors have been shown to participate in neuronal and oligodendroglial cell survival, modulation of microglial activity, and tissue repair processes [104–106]. PDGF acts as one of the key factors participating in the remission phase of MS. It promotes neuronal differentiation and remyelination, leads to increased density of oligodendrocytes, and reduces apoptosis after chronic demyelination [107–109]. PDGF also counteracts energy deprivation and oxidative stress-mediated injury [110]. It is also important for regulation and maintenance of synaptic plasticity potential, especially for LTP [111].

Few studies, both on MS patients and animal models, have investigated the role of PDGF in regard to disease course and synaptic plasticity. Mori et al. have indicated that the level of PDGF from the CSF of MS patients is positively correlated with clinical recovery [112]. Their studies showed that high PDGF concentrations were present in patients with a full recovery, whereas low PDGF levels were associated with a poor clinical outcome. As the growth factors regulate synaptic plasticity, it was also shown that CSF PDGF content correlated with the amplitude of LTP-like cortical plasticity in RR-MS patients [112]. These results were consistent with the previous* in vitro* data, showing that PDGF enhances LTP in hippocampal slices [31, 113]. High CSF concentrations of PDGF are also associated with limited clinical manifestations of new brain lesions in RR-MS [31]. However, it is worth noting that PDGF decreases with disease duration, and thus this level is low in PP-MS patients [114]. The molecular background of PDGF action on LTP is only minimally understood. Animal studies have shown that PDGF receptors are widely present in the CNS [115, 116]. Moreover, PDGF induce the expression of the* Arc/Arg3.1* gene in the hippocampus resulting in a LTP rise [113].* Arc/Arg3.1* is an immediate-early gene regulated in response to immune cell infiltration into the CNS [117]. Exposure to novelty leads to increased expression of* Arc/Arg3.1*, which is probably related to NMDA receptor activation and BDNF secretion, both of which are engaged in LTP. It was shown that* Arc/Arg3.1* levels correlate with learning performance [118]. PDGF may also exert its protective function via the inhibition of calcium overload, dependent on the NMDA receptor [119]. Excessive activation of NMDAR containing NR2B is related to excitotoxic neuronal death [120]. It is stated that PDGF facilitates LTP rather indirectly, as the level of this growth factor was similar in RR-MS patients and in healthy controls. After cTBS, healthy subjects presented with LTD rather than LTP, which is the opposite effect to what was observed for RR-MS patients after such stimulation [111].

### 3.7. The Role of Cannabinoid Type 1 Receptors (CB1Rs) in the Neural Plasticity of MS

The cannabinoid type 1 receptor (CB1R) is a G-protein coupled receptor widely distributed in the brain [121]. CB1R is expressed at the synaptic terminals of both excitatory and inhibitory neurons and regulates the release of neurotransmitters, such as GABA and glutamate [122, 123]. It is thought that the main role of CB1R-associated stimulation in MS pathogenesis is the reduction of excessive glutamate-mediated synaptic excitation and subsequent neurodegeneration [124–126].

Several studies have shown that mice with deleted CB1Rs have detrimental clinical course of EAE and low tolerance for excessive excitation resulted in neuronal damage, indicating the protective role of endogenous cannabinoids. In agreement with these findings, mice overexpressing endocannabinoids showed milder EAE course [127–131]. Indeed, it has been demonstrated that endocannabinoid anandamide (AEA) levels increase both during the acute phase of MS and in the EAE model and that elevated AEA concentrations have been able to significantly dampen the clinical and pathological outcomes of the disease [131–134]. In the striatum, CB1Rs exert a protective function on GABAergic neurons, as they limit inflammation-induced potentiation of spontaneous excitatory postsynaptic currents (sEPSCs) mediated by glutamate. CB1Rs present on GABAergic or glutamatergic neurons are differentially involved in the synaptic regulation of sEPSCs evoked by EAE. EAE mice with deleted CB1R on GABAergic neurons presented enhanced alterations in sEPSC duration, whereas mice with knock-out CB1R on glutamatergic neurons showed exacerbation in sEPSC frequency changes [135]. CB1Rs limit glutamate transmission through their binding to Gi proteins and thus through inhibition of cAMP formation [136, 137]. These results suggest that endocannabinoids regulate glutamate-mediated excitation and that both pre- and postsynaptic alterations in glutamate transmission underlie excitotoxic neurodegeneration in MS.

An alteration of the action of TNF-*α* on glutamate transmission is one possible explanation of how CB1Rs may regulate synaptic excitation [138, 139]. Studies conducted by Rossi et al. have indicated that pharmacological activation of CB1Rs reduces TNF-*α* mediated potentiation of EPSCs, which is thought to be responsible for the inflammation-induced excitotoxicity in EAE mice [138]. The antiexcitotoxic function of CB1R stimulation related to TNF-*α* is manifested by the inhibition of TNF-*α*-induced surface expression of AMPA receptors, mediating glutamate sEPSCs [140, 141].

Physical therapy (PT) was shown to have a beneficial effect on MS patients, as it resulted in enhanced synaptic transmission, nervous system remodeling and axonal sprouting, and synaptogenesis [142]. Physical activity is also an activator of endocannabinoid signaling, where CB1Rs influence synaptic plasticity. CB1Rs have been implicated in LTP regulation in MS pathogenesis, leading to motor recovery and reduced spasticity in patients after PT, but with large interpersonal differences [143, 144]. Physical therapy results in a significant upregulation of CB1R responsiveness leading to clinical amelioration from CNS damage, observed in the animal model of MS [145, 146]. Mori et al. have shown that the genetic variant of CB1R containing long AAT repeats is responsible for the poor clinical outcome after physical activity and that patients with this genetic variant do not express LTP-like cortical plasticity after TBS [147]. Reduced expression of CB1Rs triggered by a genetic defect is implicated in microglial activation in the mouse cerebellum. Such activation leads to impaired learning abilities and motor coordination due to the release of proinflammatory cytokines, mainly IL-1*β* [147, 148]. CB1Rs containing long AAT repeats may also have a negative impact on disease evolution [149, 150]. Disturbed neuronal plasticity may contribute to clinical progression, and defective CB1Rs are more frequently seen in PP-MS patients [150]. It was also evident that the genetic deletion of CB1R leads to reduced motor recovery after PT and to altered LTP in mice [151, 152]. Overall, these findings suggest that CB1Rs act as fine-tuners of glutamate transmission and excitation, as reduced expression of CB1Rs leads not only to excitotoxicity but also to altered LTP-like neuronal plasticity.

## 4. Conclusions

Our knowledge about neural plasticity has grown rapidly in the last years and continues to do so. Not long ago, the brain was considered to be an organ that slowly degenerated after a relatively long developmental stage. Currently, we know that a healthy brain uses several mechanisms, known as neural plasticity, to compensate for constant and slowly progressing neurodegeneration and to adapt to changing situations. This allows a healthy person to stay active for a very long time. The situation changes dramatically in patients with serious neurological diseases that damage the nervous system. Recent studies, however, suggest that even in those conditions the brain is still able to fight effectively in order to regain control of the body. One of these conditions is MS, a relatively common but often devastating neurological disease. The mechanisms of neural plasticity leading to the compensation of neurological symptoms are still under intensive study and are promising targets for future MS therapies.
